# Assessment of Böhme Abrasion Value of Natural Stones through Artificial Neural Networks (ANN)

**DOI:** 10.3390/ma15072533

**Published:** 2022-03-30

**Authors:** Paweł Strzałkowski, Ekin Köken

**Affiliations:** 1 Department of Mining, Faculty of Geoengineering, Mining and Geology, Wroclaw University of Science and Technology, Wybrzeże Wyspiańskiego 27, 50-370 Wrocław, Poland; 2 Nanotechnology Engineering Department, Engineering Faculty, Abdullah Gul University, Kayseri 38100, Turkey; ekin.koken@agu.edu.tr

**Keywords:** abrasion resistance, Böhme abrasion value, natural stone, artificial neural networks

## Abstract

This present study explored the Böhme abrasion value (BAV) of natural stones through artificial neural networks (ANNs). For this purpose, a detailed literature survey was conducted to collect quantitative data on the BAV of different natural stones from Turkey. As a result of the ANN analyses, several predictive models (M1–M13) were established by using the rock properties, such as the dry density (ρ_d_), water absorption by weight (w_a_), Shore hardness value (SHV), pulse wave velocity (V_p_), and uniaxial compressive strength (UCS) of rocks. The performance of the established predictive models was evaluated by using several statistical indicators, and the performance analyses indicated that four of the established models (M1, M5, M10, and M11) could be reliably used to estimate the BAV of natural stones. In addition, explicit mathematical formulations of the proposed ANN models were also introduced in this study to let users implement them more efficiently. In this context, the present study is believed to provide practical and straightforward information on the BAV of natural stones and can be declared a case study on how to model the BAV as a function of different rock properties.

## 1. Introduction

The continuous development of construction engineering generates a constant demand for building materials. In addition to the primary building materials (e.g., concrete, bricks, etc.), new or improved materials that are environmentally friendly are frequently being sought [1,2,3]. However, dimension stones and rock aggregates are among the oldest natural resources commonly used in geological, mining, and civil engineering applications. Based on modern approaches to extracting dimension stones, it has been acknowledged that the variability of natural stone quality comes from the geological, geodynamic, and geotechnical characteristics of the host rock [4,5,6]. Therefore, each natural stone has its own characteristics that should be investigated in detail. From this perspective, natural stone quality has been mainly measured through numerous laboratory testing methods. For example, the abrasion resistance of rocks is of prime importance in paving and dimension stone quality. Therefore, it is mainly quantified through several methods, such as Cerchar, Böhme, Amsler-Laffon, and Wide wheel tests [7,8,9,10,11,12,13,14,15]. Based on modern approaches to quantify the abrasivity of rocks, the Cerchar abrasivity index (CAI) has been determined, using the method suggested by Alber et al. [16]. On the other hand, the Böhme abrasion value (BAV), the Amsler–Laffon abrasion value (ALAV), and the Wide wheel abrasion value (WWA) of rocks have been determined according to EN 14157 [17]. Of the abrasion tests mentioned above, BAV is one of the most popular quantities to evaluate the quality of natural stones.

However, the BAV test is laborious and requires special equipment. In addition, Özvan and Direk [18] reported that the BAV test is expensive, long-lasting, and has negative impacts on the environment. Therefore, several relationships have been proposed to estimate the BAV of natural stones as a function of different rock properties. Nevertheless, these correlations were mainly based on simple linear and non-linear regression analysis results, considering one or two independent variables. For instance, Yaşar and Erdoğan [19] found a significant relationship between the BAV and Shore hardness value (SHV) of rocks. Similarly, Kılıç and Teymen [20] stated that the BAV of natural stones could be estimated from the SHV and pulse wave velocity (V_p_) of rocks. Teymen et al. [21] revealed strong correlations between BAV and point load strength (PLS) and SHV of rocks. Deliormanlı [22] strongly correlated the CAI with the BAV of natural stones. He also proposed two converter charts to evaluate the abrasion resistance of rocks as a function of CAI. Engin [23] investigated the cuttability of rocks by using 42 different rock types from Turkey and found a remarkable relationship between the cutting depth (CD) and BAV of considered rocks. Çobanoğlu and Çelik [24] indicated that the BAV is strongly correlated with the WWA of rocks. In addition, they also found several relationships between BAV and other rock properties, such as uniaxial compressive strength (UCS), dry unit weight (γ_d_), effective porosity (n_e_), Schmidt hammer rebound value (SHRV), SHV, and V_p_ of rocks. Bozdağ [25] also investigated the variations in BAV of 20 different rock types from Turkey as a function of the UCS, SHRV, V_p_, water absorption by weight (w_a_), n_e_, and dry density (ρ_d_) of rocks.

Based on the single and multiple regression analyses, several relationships were established to estimate the BAV as a function of the above rock properties. Bayram [26] used data-mining techniques, such as support vector machine (SVM) and random forest (RF), to estimate the BAV of different natural stones from Turkey. Based on 32 different rock types, the ρ_d_, n_e_, modulus of elasticity (E), UCS, tensile strength (TS), SHV, and PLS of the rocks were effectively used in modeling the BAV of these rocks. Recently, Mohammed et al. [27] also established several predictive models based on 22 different rocks to estimate the BAV of natural stones as a function of γ_d_, n_e_, and UCS of the rocks.

Some empirical relationships to evaluate the BAV of different rock types are listed in Table 1. Accordingly, it can be claimed that most of the physical and mechanical rock properties could be used to estimate the BAV of natural stones. Although regression-based relationships to estimate any rock property can be practical and easy to understand, they are mainly valid for small-scale datasets and, therefore, can have some limitations when dealing with broader datasets. Additionally, these usually consider single rock properties (e.g., UCS, w_a_, etc.). In this direction, it is logical to suppose that soft computing methods, which can handle a large number of datasets much easier than regression-based ones, should be attempted to provide more general empirical formulae to assess the BAV of natural stones.

In contrast to traditional computing methods, soft computing deals with approximate models and gives reliable solutions to complex problems in various engineering fields [27]. Nowadays, soft computing methods are widely used in many areas of science. In the literature, one can see numerous scientific papers that have used soft computing methods in mining [28,29,30,31] and engineering geology [32,33,34,35,36,37]. However, in terms of determining the relationship between natural stone properties, noticing such works is difficult. This work aims to present more comprehensive empirical formulations for evaluating the BAV of rocks based on soft computing methods. This novel approach presents much more reliable empirical formulations that consider multiple independent variables, while incorporating many datasets. Empirical models for BAV assessment, which are present in the literature, appear to be less flexible and comprehensive, as they assess the abrasion of a stone based on one different property of the stone that does not necessarily represent the actual abrasion process of the stone. Considering several rock properties as independent variables, the BAV may be evaluated more effectively. The use of soft computing tools to quantify natural stone quality or modeling a rock property by measuring the natural stone quality is essential for critical stones in limited resources and high demand. Using empirical formulas to assess rock BAV also eliminates the need for long-term and complex laboratory tests.

Within this context, a detailed literature survey was conducted to compile a large number of datasets, which were documented for different rocks that were used for cladding, flooring, and facade purposes in Turkey. The BAV of these natural stones was investigated through artificial neural networks (ANNs) based on different rock properties. As a result of the ANN analyses, several predictive models were established. The performance of the established models was evaluated by using several statistical indices. Given the statistical performance indices, six different predictive models were proposed to evaluate the BAV of the rock types investigated. Explicit mathematical formulations of the proposed models were also introduced to let users implement them more efficiently.

## 2. Data Documentation and Methods

Compiling datasets for the ANN analyses was based on a comprehensive literature survey. Consequently, the different datasets considered in this study are listed in Table 2. Based on this table, it is clearly seen that the ρ_d_, w_a_, SHV, V_p_, and UCS values are mainly considered to evaluate the BAV of different natural stones. Although there is a great deal of works in the literature on the BAV of natural stones, there are limited datasets available for detailed analyses. Therefore, it is necessary to collect all possible datasets to obtain more comprehensive predictive models, which can be used to estimate the BAV of natural stones. From this perspective, Özvan and Direk [18] considered the WWA and AIV as independent variables for the evaluation of BAV. In addition, Yaşar and Erdoğan [19], Kılıç and Teymen [20], Teymen and Kılıç [21], and Çobanoğlu and Çelik [22] adopted the SHV as an important rock property to estimate the BAV of natural stones. The UCS values were also adopted in the previous literature to assess the BAV of different natural stones [24,25,38]. In this study, the rock properties of ρ_d_, w_a_, SHV, V_p_, and UCS were adopted with different combinations to build such predictive models that can be used to estimate the BAV of different natural stones.

It is clearly seen from Table 2 that some of the rock properties were not reported (NR) in related documents. Therefore, it is logical to suppose that the BAV should be investigated based on different subdivided datasets. In this way, different rock properties can be included in the ANN analyses that pave the way for comparing the performance of different models adopting different rock properties.

In this manner, the documented database (Table 2) was divided into different subgroups in terms of different rock properties (i.e., ρ_d_, w_a_, SHV, V_p_, and UCS) to evaluate the BAV of natural stones deeply. The database was divided into subgroups, focusing on the independent variables available for ANN analyses. Consecutively, 13 different subdivided datasets (Set 1 to Set 13) were considered in this study (Table 3). Based on these subdivided datasets, detailed ANN analyses were performed.

## 3. Artificial Neural Network (ANN) Analyses

An artificial neural network (ANN) is a biologically inspired computational model that imitates the human brain. The applicability of ANN in engineering fields has been confirmed in that complex datasets can be modeled by using such ANN methodologies [61,62,63]. In practical ANN applications, neural networks have been trained by a feedforward backpropagation algorithm [64] to establish empirical formulae based on the weights and biases extracted from neural network analyses. In this study, the neural network toolbox (nntool) was used to establish several neural networks in the MATLAB environment.

**Figure 1:**
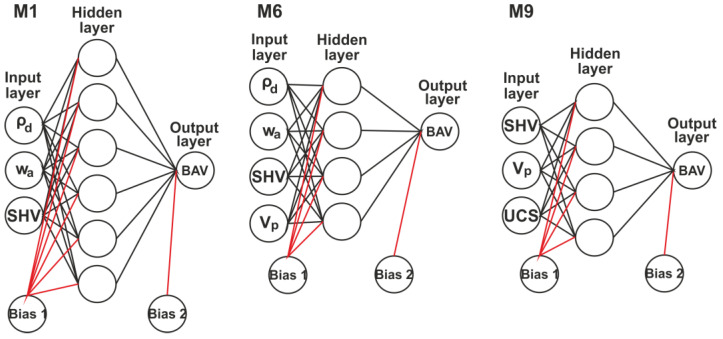
Typical ANN architectures adopted in this study (M1, Model 1; M6, Model 6; and M9, Model 9).

For this purpose, the subdivided datasets were randomly divided into training (70%) and testing/validating (30%) parts (the division is according to the commonly accepted standards in the ANN methodology). Various ANN network architectures, hidden layers, and neurons were attempted to determine the most suitable and practical structural combination. Typical ANN architectures adopted in this study are illustrated in Figure 1.

Before performing the ANN analyses, the predefined datasets (Table 3) were normalized by using Equation (1) to increase the training efficiency [65,66]. The normalization process is also essential to overcome the problems that arise from overfitting.

(1) $$V_{N}=2\left(\frac{x_{i}-x_{\min}}{x_{\max}-x_{\min}}\right)-1$$

where *x_i_* is the relevant parameter to be normalized, and *x*_min_ and *x*_max_ are the minimum and maximum values in the dataset (Table 3).

The neural network training was performed by using a feedforward backpropagation algorithm with the Levenberg–Marquardt training function. Once the ANN analyses were trained, the predictive equations could be established by using the weights and biases extracted from each ANN analysis. In this regard, predictive models for estimating the BAV of natural stones were derived by using Equation (2) [67,68]:

(2) $$y_{i}=f_{0}\left\{W_{0}\left[f_{i}\left(W_{i}\times x_{i}+B_{i}\right)\right]+B_{0}\right\}$$

where *W*_0_ and *W_i_* are the weight vectors of the output and input layers, respectively; *B*_0_ and *B_i_* are the bias vectors of the output and input layers, respectively; *x_i_* is the normalized input parameter; and *f*_0_ and *f_i_* are the transfers functions (tansig).

## 4. Results and Discussion

The correlations between the adopted rock properties and the BAV of natural stones were revealed by Pearson’s correlation coefficient (r) and Spearmen’s rho values (Table 4). Consequently, the parameters considered have different effects on the BAV of natural stones. More profoundly, the ρ_d_, SHV, V_p_, and UCS of natural stones negatively correlate with the BAV, while w_a_ has a positive correlation. Since the adopted rock properties are moderately correlated with the BAV of natural stones, they were regarded in ANN analyses with several combinations.

The performance of established predictive models was evaluated based on several statistical indices, such as the correlation of determination (R^2^), root means squared error (RMSE), and variance accounted for (VAF). The above performance indices were calculated by using Equations (3)–(5):

(3) $$R^{2}={\left(\frac{n\sum xy-\sum x\sum y}{\sqrt{n\sum x^{2}-{\left(\sum x\right)}^{2}}\sqrt{n\sum y^{2}-{\left(\sum y\right)}^{2}}}\right)}^{2}$$

(4) $$\mathrm{RMSE}=\sqrt{\frac{\sum_{i=1}^{n}{\left(y_{i}-x_{i}\right)}^{2}}{n}}$$

(5) $$\mathrm{VAF}=\left(1-\frac{\mathrm{var}\left(y_{i}-x_{i}\right)}{\mathrm{var}(y_{i})}\right)\times 100$$

where *y* is the observed data, *x* is the estimated data, and *n* is the number of datasets.

The performance evaluation of the established models is presented in Table 5. Higher R^2^ and VAF and lower RMSE values indicate relatively more successful models. In this direction, one can notice from Table 5 that the performance of the predictive models is quite different due to the different combinations in rock properties and various ANN architectures. Nevertheless, ANN analysis results are indicated to be more effective in assessing BAV than simple correlations between the BAV and adopted rock properties.

For the established predictive models, the R^2^, RMSE, and VAF values were found to be between 0.68 and 0.97, 3.260 and 10.111, and 59.78 and 96.81, respectively. It can also be claimed that the variations in the number of datasets (*n*) related to the different input parameters can also be an essential parameter in the performance of the predictive models. For example, the best R^2^ values among the established predictive models were found for the M6 and M9 models (R^2^ ≥ 0.96). For these models, the number of datasets was 48 and 67, respectively. For other models (e.g., M1 and M10), the number of datasets was more than those, which can also affect the R^2^ values of these models. Therefore, further studies considering the same length datasets with adopting the same or different rock properties may be beneficial.

The ANN analysis results also indicated that the quantitative evaluations on BAV should be performed by adopting at least two—preferably three or four rock properties—to obtain more successful predictive models. Adopting more rock properties to establish a predictive model also illustrates the most realistic abrasion process. In addition, it should be mentioned that different combinations of rock properties seem to affect the number of hidden layers in the ANN analyses.

**Figure 2:**
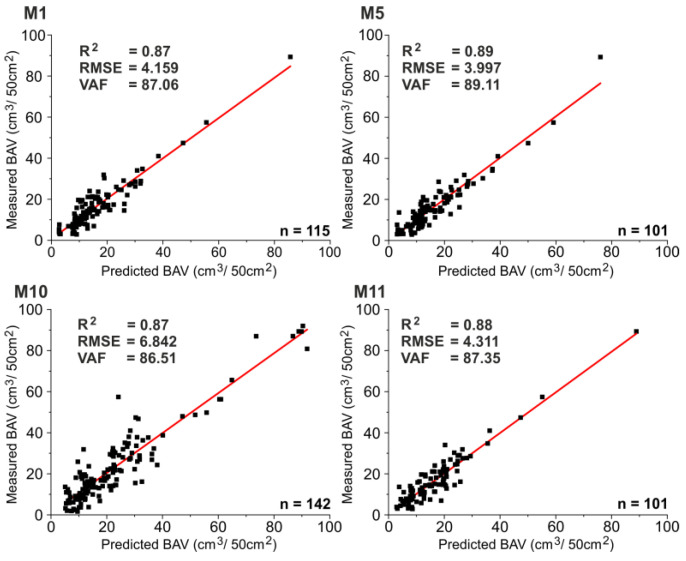
Predicted and measured BAV values for the proposed ANN models.

Among the models of M1–M13, those with R^2^ greater than 0.85 were selected due to the high fit of the analyzed data. Additionally, although their R^2^ values are greater than 0.96, for better reliability, the models of M6 and M9 were not proposed as reliable tools to assess the BAV of natural stones, due to having small-scale datasets. It should be mentioned that a large number of input data enables the prediction of models with a higher capability to estimate the BAV. Of the established predictive models, M1, M5, M10, and M11 (Table 5) can be declared feasible approaches to estimate the BAV of natural stones. For these models, the predicted and measured BAV values are plotted in Figure 2.

Figure 2 shows that the predicted and measured BAV values are in good agreement. However, by focusing on different rock properties with several combinations, further studies can be beneficial in evaluating the BAV of natural stones. Herein, the effects of the different number of datasets and hidden layers should also be considered in future ANN models.

Sensitivity analyses were also performed to determine which input parameter is more influential in the proposed ANN models. In this study, the cosine amplitude method (CAM) was used to assess the sensitivity of each input parameter used in the ANN analyses. Several researchers [69,70,71,72,73] also adopted this method (see Equation (6)) to evaluate the sensitivity degree of each input parameter by determining the correlation degree (*r_ij_*) between the input and output pairs. The higher the value of *r_ij_*, the greater is the effect of the relevant input parameter.

(6) $$r_{ij}=\frac{\sum_{i=1}^{n}\left(x_{i}y_{i}\right)}{\sqrt{\sum_{i=1}^{n}{\left(x_{i}\right)}^{2}\sum_{i=1}^{n}{\left(y_{i}\right)}^{2}}}$$

where *x_i_* is the input parameter, *y_i_* is the output parameter, and *n* is the number of datasets used in the analysis.

**Figure 3:**
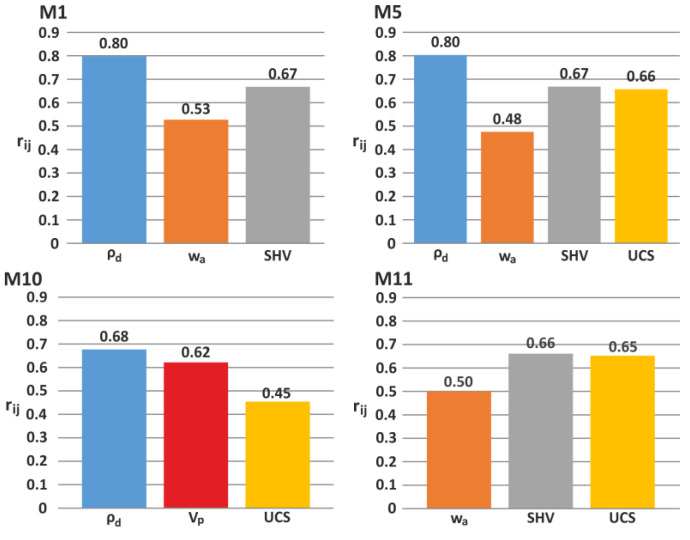
Sensitivity analysis results of the proposed ANN models.

Based on the sensitivity analysis results (Figure 3), it was determined that the ρ_d_ is more influential for M1, M5, and M10. For these models, the *r_ij_* of ρ_d_ ranged from 0.68 to 0.80. For the other proposed model, M11, the effects of w_a_ (*r_ij_* = 0.50), UCS (*r_ij_* = 0.65), and SHV (*r_ij_* = 0.66) are mainly lower than those of the other parameters included in the other models. To sum up, the sensitivity analyses demonstrated that, when input parameters are changed, their effects are also changed during the training of ANN models.

Last but not least, the empirical formulae of the proposed ANN models and their sub-equation systems are listed in Table 6 and Table 7, respectively. Therefore, the ANN models stated in this study can be easily implemented by coding the given equations in any computational language. In this way, the BAV of natural stones can be elaborately assessed with respect to different rock properties. In this context, the present study can be declared a case study on modeling the BAV of different rock types by using different ANN models. Furthermore, these models can be reliably used to estimate the BAV of natural stones without using abrasive powders, negatively affecting people who perform the BAV test in the laboratory.

## 5. Conclusions

The present study encompassed a comprehensive literature survey to evaluate the BAV of different natural stones from Turkey. It was observed during the literature survey that most previous studies to assess the BAV are based on regression analyses. In these analyses, various rock properties with small-scale datasets were considered to estimate the BAV of different natural stones. In this study, the BAV of different natural stones was investigated by using ANN analyses based on relatively large-scale datasets. Based on the collected data, 13 different subdivided datasets were created for the ANN analyses. In these analyses, different rock properties, such as ρ_d_, w_a_, SHV, V_p_, and UCS, were considered. As a result of the ANN analyses, 13 different predictive models (M1–M13) were established in this study.

The performance of the established predictive models was evaluated by using several statistical indicators. In light of these indicators, four different predictive models (M1, M5, M10, and M11) were proposed to estimate the BAV of natural stones. These models provide promising results when comparing the predicted and measured BAV values. Furthermore, the sensitivity analyses revealed the effectiveness of the input parameters in the proposed ANN models. Consequently, different rock properties become prominent when the model architecture changes. Explicit mathematical formulations of the proposed ANN models were also introduced to let users implement the proposed models more efficiently.

This work demonstrated that the BAV could be predicted reliably from some physical and mechanical rock properties. Models for BAV assessment allow for the avoidance of long-term and complex laboratory tests, which additionally cause damage to the stone during the abrasion process. The present study, in this context, provides practical and straightforward knowledge about the BAV of natural stones and can be successfully used for modeling the BAV as a function of different rock properties.

## Figures and Tables

**Table 1:** Regression-based models to evaluate the BAV of natural stones.

Independent Variable	Rock Type	Number of Datasets, *n*	Empirical Formula	R^2^	Reference
WWA (mm)	Basalt, Granite, Limestone, Travertine, İgnimbrite	13	BAV=3.057WWA−53.607 *	0.92	[18]
AIV (%)			BAV=2.516AIV−45.086 *	0.91	
SHV (−)	Limestone, Marble, Basalt, Sandstone	6	BAV=−1.2363SHV+94.648	0.66	[19]
SHV (−)	Diorite Quartzite Sandstone Granodiorite Basalt Limestone Trachyte Travertine Andesite, Tuff Marble	19	$\mathrm{BAV}=10553{\mathrm{SHV}}^{-1.6868}$	0.92	[20]
V_p_ (km/s)			$\mathrm{BAV}=579.97V_{p}{}^{-2.4279}$	0.85	
PLS (MPa)			$\mathrm{BAV}=69.578{\mathrm{PLS}}^{-1.4807}$	0.76	
SHRV (−)			$\mathrm{BAV}=136910{\mathrm{SHRV}}^{-2.3621}$	0.91	
n_e_ (%)			$\mathrm{BAV}=8.935\exp\left(0.0857n_{e}\right)$	0.89	
PLS (MPA)	Marble, Travertine	14	BAV=50.685exp(−0.2134PLS)	0.85	[21]
SHV (−)			BAV=112.87exp(−0.043SHV)	0.75	
CAI (−)	Marble	15	BAV=−4.64CAI+25.06	0.83	[22]
CD (mm)	Marble, Limestone, Sandstone, Travertine, Granite, Andesite, Diabase, Tuff, Marl	42	BAV=11.574ln(CD)−25.417 *	0.78	[23]
WWA (mm)	Limestone, Travertine, Dolomite, Granite, Marble, Andesite, Serpentine, Latite, Autoclaved Aerated Concrete, Briquette	32	BAV=5.192WWA−81.4333 *	0.94	[24]
γ_d_ (kN/m^3^)			$\mathrm{BAV}=-7.8496\gamma_{d}+223.5$	0.81	
n_e_ (+)			$\mathrm{BAV}=4.8095n_{e}+12.046$	0.83	
SHRV (−)			BAV=−2.1805SHRV+139.22	0.39	
SHV (−)			BAV=143.14exp(−0.039SHV)	0.70	
V_p_ (km/s)			$\mathrm{BAV}=-25.577V_{p}+181.91$	0.54	
UCS (MPa)			BAV=−37.17ln(UCS)+193.7	0.70	
UCS (MPa)	Tuff, Andesite, Granite, Marble, Dolomite, Travertine	20	BAV=−10.798ln(UCS)+57.199	0.89	[25]
SHRV (−)			BAV=−23.274ln(SHRV)+95.272	0.85	
w_a_ (%)			$\mathrm{BAV}=0.028w_{a}{}^{2}+0.346w_{a}+8.951$	0.41	
V_p_ (km/s)			$\mathrm{BAV}=3.419V_{p}{}^{2}-31.398V_{p}+83.364$	0.77	
ρ_d_ (g/cm^3^)			$\mathrm{BAV}=13.997\rho_{d}{}^{2}-75.882\rho_{d}+110.675$	0.45	
γ_d_ (kN/m^3^)	Tuff, Andesite, Basalt, sandstone, limestone	22	$\mathrm{BAV}=-35.43\ln\left(\gamma_{d}\right)+116.06$	0.88	[26]
w_a_ (%)			$\mathrm{BAV}=1.0408w_{a}+0.5077$	0.94	
UCS (MPa)			$\mathrm{BAV}=1378.4{\mathrm{UCS}}^{-1.766}$	0.65	

Explanations: BAV, Böhme abrasion value (cm^3^/50cm^2^); γ_d_, dry unit weight; w_a_, water absorption by weight; UCS, uniaxial compressive strength; PLS, point load strength; SHV, Shore hardness value; CD, cutting depth; SHRV, Schmidt hammer rebounding value; V_p_, pulse wave velocity; ρ_d_, dry density; WWA, wide wheel abrasion value; AIV, aggregate impact value; n_e_, effective porosity; CAI, Cerchar abrasivity index. * The empirical formula was modified by reversing the original one.

**Table 2:** Datasets adopted in this study.

ρ_d_ (g/cm^3^)	w_a_ (%)	SHV (−)	V_p_ (km/s)	UCS (MPa)	BAV (cm^3^/50cm^2^)	*n*	Reference
1.33–3.07	0.19–27.41	NR	1.88–6.17	11.65–150.68	5.58–87.02	13	[18]
2.52–2.72	NR	53.05–63.09	NR	40.10–111.51	13.25–28.25	6	[19]
NR	NR	11.00–82.00	1.47–6.75	6.20–239.00	5.00–181.60	19	[20]
2.36–2.70	0.10–2.09	40.70–66.50	NR	NR	6.21–20.30	14	[21]
1.51–2.93	0.02–17.35	14.60–110.20	NR	13.60–256.40	3.05–28.58	42	[23]
2.23–2.80	0.09–4.34	21.70–73.50	4.55–7.14	32.37–253.97	6.83–89.32	30	[24]
1.41–2.81	0.27–24.43	NR	2.03–6.03	10.50–188.13	1.62–35.11	20	[25]
2.10–2.71	NR	36.00–67.00	NR	42.00–126.80	6.84–27.70	32	[26]
2.76–2.86	0.04–0.15	36.98–51.65	NR	67.70–159.21	18.01–34.01	12	[39]
1.40	23.00	NR	1.80	9.00	48.00	1	[40]
2.59–2.76	0.14–3.40	NR	NR	62.40–65.00	18.35–30.48	2	[41]
2.55–2.80	0.61–2.91	NR	NR	90.20–93.40	21.70–25.50	2	[42]
2.65–2.73	0.03–1.57	49.56–65.14	4.94–6.47	50.70–169.80	2.89–14.51	18	[43]
2.70	0.18	NR	5.92	NR	18.47	1	[44]
1.25–2.68	0.32–28.23	NR	2.02–6.21	7.57–141.56	5.21–46.74	22	[45]
1.34–2.68	0.11–25.51	NR	1.33–5.21	5.84–59.90	14.55–80.85	17	[46]
2.69	0.22	NR	6.47	109.70	8.86	1	[47]
2.72–2.75	0.10–0.90	NR	NR	61.20–184.70	10.30–24.60	8	[48]
2.71	0.11	NR	5.64	81.80	10.28	1	[49]
2.61	1.29	NR	5.96	99.00	9.13	1	[50]
1.09–1.73	13.26–39.34	NR	1.80–3.00	2.75–87.50	15.50–92.00	9	[51]
2.63–2.67	0.87–1.81	NR	5.22–5.83	121.60–158.40	6.12–7.47	3	[52]
2.72	0.02	NR	NR	100.40	11.01	1	[53]
2.84	0.22	NR	5.11	179.40	12.43	1	[54]
2.62	0.42	90.80	NR	206.13	7.64	1	[55]
2.74	0.16	57.20	NR	69.84	11.80	1	[56]
2.71	0.25	NR	NR	69.55	12.65	1	[57]
2.69–2.70	0.19–0.22	NR	4.73–6.07	72.35–97.00	10.55–15.02	3	[58]
2.60	0.81	NR	4.27	117.08	20.57	1	[59]
2.14–2.72	0.06–5.05	NR	5.25–6.40	57.10–110.70	8.59–27.70	4	[60]

Explanations: ρ_d_, dry density; w_a_, water absorption by weight; SHV, Shore hardness value; V_p_, pulse wave velocity; UCS, uniaxial compressive strength; BAV, Böhme abrasion value; *n*, number of samples; NR, not reported.

**Table 3:** Subdivided datasets for ANN analyses.

Dataset No.	Independent Variable	Number of Datasets, *n*	Additional Information
Set 1	ρ_d_, w_a_, SHV	115	ρ_d_ = 1.510–2.929 g/cm^3^ w_a_ = 0.023–17.35% SHV = 14.60–110.20 BAV = 2.89–89.32 cm^3^/50cm^2^
Set 2	ρ_d_, w_a_, V_p_	145	ρ_d_ = 1.087–3.070 g/cm^3^ w_a_ = 0.023–39.34% V_p_ = 1.33–7.14 km/s BAV = 1.62–92.00 cm^3^/50cm^2^
Set 3	ρ_d_, w_a_, UCS	213	ρ_d_ = 1.087–3.070 g/cm^3^ w_a_ = 0.023–39.34% UCS = 2.75–256.40 MPa BAV = 1.62–92.00 cm^3^/50cm^2^
Set 4	ρ_d_, w_a_	230	ρ_d_ = 1.087–3.070 g/cm^3^ w_a_ = 0.023–39.34% BAV= 1.62–92.00 cm^3^/50cm^2^
Set 5	ρ_d_, w_a_, SHV, UCS	101	ρ_d_ = 1.510–2.929 g/cm^3^ w_a_ = 0.023–17.35% SHV = 14.60–110.20 UCS = 13.60–256.40 MPa BAV = 2.89–89.32 cm^3^/50cm^2^
Set 6	ρ_d_, w_a_, SHV, V_p_	48	ρ_d_ = 2.222–2.797 g/cm^3^ w_a_ = 0.023–4.34% SHV = 21.70–73.50 V_p_ = 4.55–7.14 km/s BAV = 2.89–89.32 cm^3^/50cm^2^
Set 7	w_a_, V_p_	145	w_a_ = 0.023–4.34% V_p_ = 1.33–7.14 km/s BAV = 1.62–92.00 cm^3^/50cm^2^
Set 8	ρ_d_, UCS	251	ρ_d_ = 1.087–3.070 g/cm^3^ UCS = 2.75–256.40 MPa BAV = 1.62–92.00 cm^3^/50cm^2^
Set 9	SHV, V_p_, UCS	67	SHV = 11.00–82.00 V_p_ = 1.47–7.14 km/s UCS = 6.20–253.97 MPa BAV = 2.89–181.6 cm^3^/50cm^2^
Set 10	ρ_d_, V_p_, UCS	142	ρ_d_ = 1.087–3.070 g/cm^3^ V_p_ = 1.33–7.14 km/s UCS = 2.75–253.97 MPa BAV = 1.62–92.00 cm^3^/50cm^2^
Set 11	w_a_, SHV, UCS	101	w_a_ = 0.023–17.35% SHV = 14.60–110.20 UCS = 13.60–256.40 MPa BAV = 2.89–89.32 cm^3^/50cm^2^
Set 12	w_a_, SHV	115	w_a_ = 0.023–17.35% SHV = 14.60–110.20 BAV = 2.89–89.32 cm^3^/50cm^2^
Set 13	w_a_, UCS	213	w_a_ = 0.023–39.34% UCS = 2.75–256.40 Mpa BAV = 1.62–92.00 cm^3^/50cm^2^

**Table 4:** Correlations between the considered rock properties and BAV of natural stones.

Parameter	BAV		Number of Datasets, *n*
	Pearson’s Correlation Coefficient, r	Spearman’s Rho	
ρ_d_	−0.589	−0.366	268
w_a_	0.674	0.469	230
SHV	−0.603	−0.742	172
V_p_	−0.529	−0.512	164
UCS	−0.531	−0.680	270

**Table 5:** Performance evaluation of the ANN-based predictive models.

Model No.	ANN Architecture	Independent Variables	Number of Datasets, *n*	R^2^	RMSE	VAF
**M1**	**3–6–1**	**ρ_d_, w_a_, SHV**	**115**	**0.87**	**4.159**	**87.06**
M2	3–10–1	ρ_d_, w_a_, V_p_	145	0.80	8.202	80.23
M3	3–9–1	ρ_d_, w_a_, UCS	213	0.79	7.591	78.57
M4	2–14–1	ρ_d_, w_a_	230	0.60	9.972	59.78
**M5**	**4–6–1**	**ρ_d_, w_a_, SHV, UCS**	**101**	**0.89**	**3.997**	**89.11**
M6	4–4–1	ρ_d_, w_a_, SHV, V_p_	48	0.96	3.260	95.56
M7	2–8–1	ρ_d_, V_p_	145	0.71	10.111	69.97
M8	2–10–1	ρ_d_, UCS	251	0.68	8.561	68.01
M9	3–4–1	SHV, V_p_, UCS	67	0.97	5.626	96.81
**M10**	**3–10–1**	**ρ_d_, V_p_, UCS**	**142**	**0.87**	**6.842**	**86.51**
**M11**	**3–6–1**	**w_a_, SHV, UCS**	**101**	**0.88**	**4.311**	**87.35**
M12	2–10–1	w_a_, SHV	115	0.84	4.643	83.76
M13	2–12–1	w_a_, UCS	213	0.69	9.139	68.26

Note: Bolded models (e.g., **M5**) were proposed to evaluate the BAV in this study.

**Table 6:** Empirical formulae of the proposed ANN models.

Model No.	Empirical Formula	R^2^
M1	$\mathrm{BAV}=43.215\tanh\left(\sum_{i=1}^{6}A_{i}-0.33543\right)+46.105$	0.87
M5	$\mathrm{BAV}=43.215\tanh\left(\sum_{i=1}^{6}B_{i}+3.4889\right)+46.105$	0.89
M10	$\mathrm{BAV}=45.19\tanh\left(\sum_{i=1}^{10}E_{i}+1.0233\right)+46.81$	0.87
M11	$\mathrm{BAV}=43.215\tanh\left(\sum_{i=1}^{6}F_{i}-4.1292\right)+46.105$	0.88

**Table 7:** Sub-equation systems of the proposed ANN models.

Model 1, M1
A1=12.4623tanh(−6.9726ρdn−1.0128wan+6.5392SnHV+5.7867)
A2=−13.0293tanh(−6.6434ρdn−1.2408wan+6.3398SnHV+5.275)
A3=0.66333tanh(41.9403ρdn+25.957wan+46.7092SnHV+28.4786)
A4=1.0456tanh(−8.1555ρdn−4.4647wan−0.53822SnHV−1.1943)
A5=−0.68409tanh(−8.3198ρdn−0.22724wan+5.2452SnHV+2.5517)
A6=0.82104tanh(−2.6995ρdn+4.0335wan−0.66263SnHV−2.7921)
Normalization functions
ρdn=1.4085ρd−3.1268 wan=0.1154wa−1.0023 SnHV=0.0209SHV−1.3054
**Model 5, M5**
B1=−8.443tanh(0.1734ρdn+0.35274wan+0.92719SnHV−0.27225UnCS+1.2055)
B2=5.0854tanh(15.6798ρdn+11.9027wan−7.1636SnHV+7.4804UnCS+0.81768)
B3=−3.6021tanh(0.92606ρdn+6.4781wan+0.57942SnHV−9.3195UnCS−1.6663)
B4=5.4555tanh(−13.1733ρdn−9.3031wan+6.9893SnHV−6.6755UnCS+0.08943)
B5=−3.1808tanh(−0.77901ρdn−7.9121wan−2.0084SnHV+10.0399UnCS+0.31293)
B6=−1.0385tanh(−15.4378ρdn−10.435wan−7.4539SnHV+12.2823UnCS−3.7345)
Normalization functions
ρdn=1.4094ρd−3.1283 wan=0.1154wa−1.0027
SnHV=0.0209SHV−1.3054 UnCS=0.0082UCS−1.112
**Model 10, M10**
$E_{1}=-0.78948\tanh\left(0.45826^{n}\rho_{d}-1.5562^{n}V_{p}+1.4814^{n}\mathrm{UCS}-4.0742\right)$
$E_{2}=-7.3318\tanh\left(3.6404^{n}\rho_{d}+6.231^{n}V_{p}-1.9816^{n}\mathrm{UCS}+6.2999\right)$
$E_{3}=-6.7869\tanh\left(-16.7298^{n}\rho_{d}-8.2141^{n}V_{p}-4.4633^{n}\mathrm{UCS}-5.9214\right)$
$E_{4}=7.3084\tanh\left(-1.922^{n}\rho_{d}+1.1762^{n}V_{p}-2.2753^{n}\mathrm{UCS}+0.2467\right)$
$E_{5}=-9.8086\tanh\left(-3.1249^{n}\rho_{d}+1.044^{n}V_{p}-2.4611^{n}\mathrm{UCS}+0.81496\right)$
$E_{6}=-6.664\tanh\left(22.0246^{n}\rho_{d}-3.5822^{n}V_{p}+10.4282^{n}\mathrm{UCS}+6.7415\right)$
$E_{7}=-0.27279\tanh\left(-2.6309^{n}\rho_{d}+19.0258^{n}V_{p}-16.2483^{n}\mathrm{UCS}-2.2838\right)$
$E_{8}=-3.4797\tanh\left(6.0836^{n}\rho_{d}-0.54222^{n}V_{p}+2.5712^{n}\mathrm{UCS}-2.5026\right)$
$E_{9}=13.4292\tanh\left(12.7002^{n}\rho_{d}+9.0008^{n}V_{p}-11.4326^{n}\mathrm{UCS}+5.3285\right)$
$E_{10}=-9.0046\tanh\left(20.3148^{n}\rho_{d}+9.631^{n}V_{p}-15.7653^{n}\mathrm{UCS}+6.8938\right)$
Normalization functions
${}^{n}\rho_{d}=1.0101\rho_{d}-2.101\;$ ${}^{n}V_{p}=0.3442V_{p}-1.4578\;$ ${}^{n}\mathrm{UCS}=0.008\mathrm{UCS}-1.0219$
**Model 11, M11**
$F_{1}=3.3586\tanh\left(2.7968^{n}w_{a}-3.2106^{n}\mathrm{SHV}-1.7192^{n}\mathrm{UCS}-2.6267\right)$
$F_{2}=2.8111\tanh\left(-6.9362^{n}w_{a}-3.2793^{n}\mathrm{SHV}+7.9886^{n}\mathrm{UCS}-2.4465\right)$
$F_{3}=-3.2889\tanh\left(-4.5788^{n}w_{a}-1.7085^{n}\mathrm{SHV}+6.7829^{n}\mathrm{UCS}-0.61604\right)$
$F_{4}=3.8739\tanh\left(-6.6335^{n}w_{a}-0.0497^{n}\mathrm{SHV}-1.9314^{n}\mathrm{UCS}-3.7471\right)$
$F_{5}=-0.63548\tanh\left(-4.5811^{n}w_{a}+4.4011^{n}\mathrm{SHV}-0.13051^{n}\mathrm{UCS}-3.8339\right)$
$F_{6}=-3.1414\tanh\left(-1.7301^{n}w_{a}-0.48243^{n}\mathrm{SHV}-2.7502^{n}\mathrm{UCS}-4.4435\right)$
Normalization function
${}^{n}w_{a}=0.1154w_{a}-1.0023\;$ ${}^{n}\mathrm{SHV}=0.0209\mathrm{SHV}-1.3054\;$ ${}^{n}\mathrm{UCS}=0.0082\mathrm{UCS}-1.1121$

## Data Availability

Not applicable.
